# Hypoxic Conditions Promote the Angiogenic Potential of Human Induced Pluripotent Stem Cell-Derived Extracellular Vesicles

**DOI:** 10.3390/ijms22083890

**Published:** 2021-04-09

**Authors:** André Cronemberger Andrade, Martin Wolf, Heide-Marie Binder, Fausto Gueths Gomes, Felix Manstein, Patricia Ebner-Peking, Rodolphe Poupardin, Robert Zweigerdt, Katharina Schallmoser, Dirk Strunk

**Affiliations:** 1 Cell Therapy Institute, Spinal Cord Injury and Tissue Regeneration Center Salzburg (SCI-TReCS), Paracelsus Medical University (PMU), 5020 Salzburg, Austria; andre.cronemberger@pmu.ac.at (A.C.A.); martin.wolf@pmu.ac.at (M.W.); heide.binder@pmu.ac.at (H.-M.B.); patricia.ebner@pmu.ac.at (P.E.-P.); rodolphe.poupardin@pmu.ac.at (R.P.); 2 Department of Transfusion Medicine and SCI-TReCS, Paracelsus Medical University (PMU), 5020 Salzburg, Austria; fausto.gueths@pmu.ac.at (F.G.G.); k.schallmoser@salk.at (K.S.); 3 Department of Cardiac, Thoracic, Transplantation and Vascular Surgery, Leibniz Research Laboratories for Biotechnology and Artificial Organs (LEBAO), Hannover Medical School, 30625 Hannover, Germany; manstein.felix@mh-hannover.de (F.M.); Zweigerdt.Robert@mh-hannover.de (R.Z.)

**Keywords:** extracellular vesicles (EV), induced pluripotent stem cells (iPSC), hypoxia, angiogenesis, regenerative medicine, hypoxia-inducible transcription factor (HIF)

## Abstract

Stem cells secrete paracrine factors including extracellular vesicles (EVs) which can mediate cellular communication and support the regeneration of injured tissues. Reduced oxygen (hypoxia) as a key regulator in development and regeneration may influence cellular communication via EVs. We asked whether hypoxic conditioning during human induced pluripotent stem cell (iPSC) culture effects their EV quantity, quality or EV-based angiogenic potential. We produced iPSC-EVs from large-scale culture-conditioned media at 1%, 5% and 18% air oxygen using tangential flow filtration (TFF), with or without subsequent concentration by ultracentrifugation (TUCF). EVs were quantified by tunable resistive pulse sensing (TRPS), characterized according to MISEV2018 guidelines, and analyzed for angiogenic potential. We observed superior EV recovery by TFF compared to TUCF. We confirmed hypoxia efficacy by HIF-1α stabilization and pimonidazole hypoxyprobe. EV quantity did not differ significantly at different oxygen conditions. Significantly elevated angiogenic potential was observed for iPSC-EVs derived from 1% oxygen culture by TFF or TUCF as compared to EVs obtained at higher oxygen or the corresponding EV-depleted soluble factor fractions. Data thus demonstrate that cell-culture oxygen conditions and mode of EV preparation affect iPSC-EV function. We conclude that selecting appropriate protocols will further improve production of particularly potent iPSC-EV-based therapeutics.

## 1. Introduction

Lifelong homeostasis and regeneration of the vascular system is of critical importance for a multiplicity of physiological and pathological processes. Sprouting angiogenesis as well as neo-vessel formation by individual vascular cells, termed vasculogenesis, play key roles in health and disease. The therapeutic potential of endothelial progenitor cells for new vessel formation was recognized already decades ago. Highly proliferative endothelial colony-forming progenitor cells (ECFCs) are capable of producing functional endothelial cells in vitro and in vivo [1]. The creation of patent vessels requires coordinated interaction of endothelial cells with perivascular cells [2,3,4]. Endothelial differentiation of induced pluripotent stem cells (iPSCs) represents a virtually unlimited source of endothelial cells for research and future therapy [5,6]. Ideally, somatic cells together with vascular endothelial cells and stromal cells functioning as pericytes can be generated from the same iPSC clone for organoid formation and development of organ regeneration strategies [7,8].

Reduced oxygen level (hypoxia) was demonstrated to induce the expression of vascular endothelial growth factor (VEGF) in ischemic tissue, during wound healing and tumor angiogenesis. Hypoxia-inducible transcription factors (HIFs) are key molecular regulators of the hypoxia response and target for pharmacological regulation of different types of angiogenesis-related pathologies ranging from inflammatory bowel and kidney disease to cancer [9]. Recent insights into the mechanisms of oxygen sensing and hypoxia signaling resulted in the development of HIF-2α-targeted therapies for patients with von Hippel-Lindau-associated tumors [10]. Regarding the role of HIFs during cell-based therapies, we and others have previously shown that hypoxia sensing by pericyte progenitors can regulate therapeutic vasculogenesis, vascular pathologies and regeneration [11,12,13]. Active crosstalk between ECFCs and pericytes within the first hours after cell transplantation was further demonstrated to be decisive towards establishing long-term stable vasculature [14].

Extracellular vesicles (EVs) represent a heterogeneous family of membrane-enclosed nanovesicles secreted via several mechanisms by virtually all cells. EVs act as mediators of intercellular communication by transferring lipids, proteins, and various nucleic acid species of the source cell in a presumably organized manner [15]. EVs are also considered as novel therapeutic and diagnostic tools due to their capacity to transfer bioactive components to recipient cells and tissues, and their contribution to intercellular communication [16,17]. EVs can support angiogenesis, tissue regeneration, participate in immune modulation, and thereby contribute to the mode of action of cellular therapies. EV investigations are challenging because they are close to or below the detection limit of many conventional analysis methods. Nanometer-sized lipoproteins and protein aggregates overlap with EVs in size or density, thus complicating EV purification and characterization methods [18]. In 2018, the International Society for Extracellular Vesicles (ISEV) therefore updated their comprehensive Minimal Information for Studies of Extracellular Vesicles (MISEV) guidelines to assure a better standardization and quality in the field [19]. Powerful attempts are also underway to translate EV research results into effective therapies [20].

The majority of studies aiming to improve re-vascularization by means of EV treatment have so far tested mature somatic stem cell-derived EVs or EVs secreted by differentiated iPSC progeny or stromal cells [21]. Limited data are available on the phenotype and function of iPSC-EVs, despite the fact that trophic paracrine factors were recognized to play a role in iPSC function [22]. Mouse iPSC-EVs were demonstrated to be superior to iPSCs, improving left ventricular function after experimental myocardial infarction [23]. Protective effects of human iPSC-derived exosomes during high glucose-induced endothelial cell injury were also observed, indicating applicability of human iPSC-EVs in diabetic angiopathy [24]. Most recently, an interactome analysis of a human iPSC secretome revealed protein interaction networks capable of inducing a pro-regenerative shift in macrophages after iPSC-EV treatment in vitro. Whether these regulatory factors were secreted as soluble proteins rather than being transported as an iPSC-EV cargo was not addressed in the study [25].

In this study, we established an experimental strategy for large-scale human iPSC secretome separation into iPSC-EVs and secreted soluble factors. We further asked if hypoxia as an established inducer of angiogenesis also influences iPSC-EV function. We found that effective hypoxic conditioning of human iPSCs resulted in the secretion of iPSC-EVs with significantly elevated angiogenic potential that were significantly more potent than corresponding soluble factors in inducing vascular network formation in vitro.

## 2. Results

### 2.1. Pluripotency Markers and Viability of iPSCs Were Maintained after Large-Scale Expansion for EV Production

Starting iPSC cultures from one million cells/well in six-well culture plates allowed for propagating 9.767 × 10^8^ ± 1.201 × 10^8^ iPSCs (mean ± SD; n = 3) per four-layered cell factory (CF4) within eight days. The conditioned medium was collected daily between days 4–8. Pooled conditioned medium (2.7 L per batch) was used for large-scale EV production.

**Figure 1:**
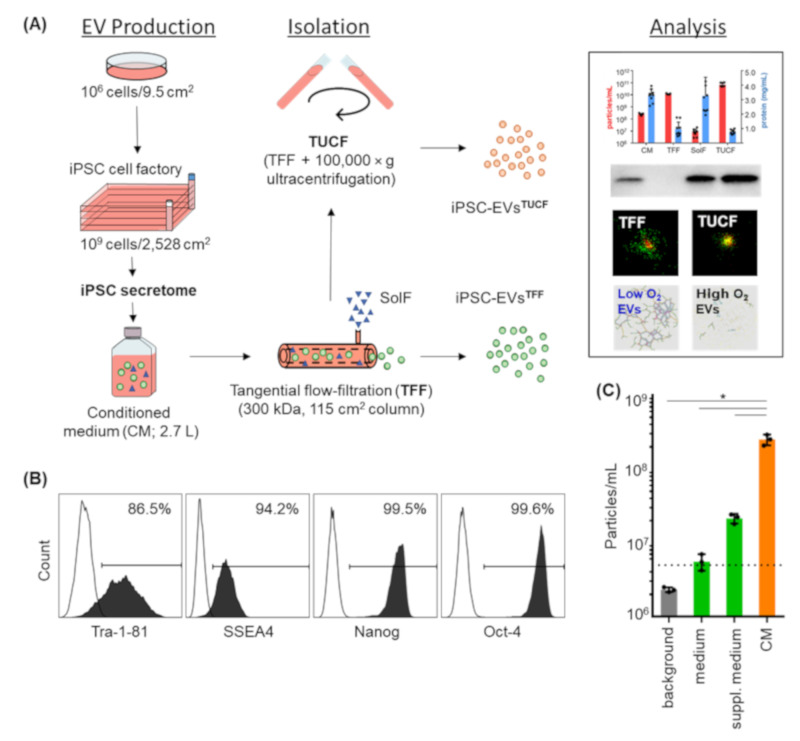
Large-scale production of induced pluripotent stem cell-derived extracellular vesicles (iPSC-EVs). (**A**) Schematic representation of large-scale EV production from iPSCs. After culture in six-well plates, iPSCs were transferred directly to T225 flasks and then to four-layered cell factories. Culture-conditioned medium (CM) was pooled and separated into soluble factors (SolF) and enriched EVs by tangential flow filtration (TFF). For further concentration and protein depletion, TFF-enriched EVs were submitted to ultracentrifugation (TUCF). Comparative EV analysis included particle, protein and EV identity determination as illustrated plus single EV phenotyping and function tests. (**B**) Identity and purity of human iPSCs determined by Tra-1-81, SSEA-4, Nanog, OCT-4 flow cytometry (representative data shown). (**C**) Particle concentration during EV production measured by tunable resistive pulse sensing (TRPS) in background (PBS + 0.05% Tween 20, 0.22 µm pore filtered), basic and supplemented defined serum-free medium (mTeSR™1) before and after culturing cells. Mean ± SEM of three independent experiments; individual results for CM are shown in Figure 2A. Dotted line represents limit of detection. Mean ± SD of three measurements (* *p* < 0.05).

EVs were separated from cell-secreted soluble factors by TFF and concentrated to 10 mL final volume per batch. Subsequent ultracentrifugation for further particle enrichment was done in selected experiments as indicated (Figure 1A). The iPSCs appeared morphologically immature during large-scale expansion with a viability > 90%. Flow cytometry confirmed homogenous Tra-1-81, SSEA4, Nanog and Oct4 pluripotency marker expression (Figure 1B). To confirm that the defined medium was suitable for iPSC-EV analysis we evaluated the particle concentration by TRPS showing 2.153 × 10^7^ ± 0.319 × 10^7^ particles (mean ± SD) in supplemented medium in advance of initiating cell culture (Figure 1C).

### 2.2. Monitoring Particle Recovery during Subsequent Purification

**Figure 2:**
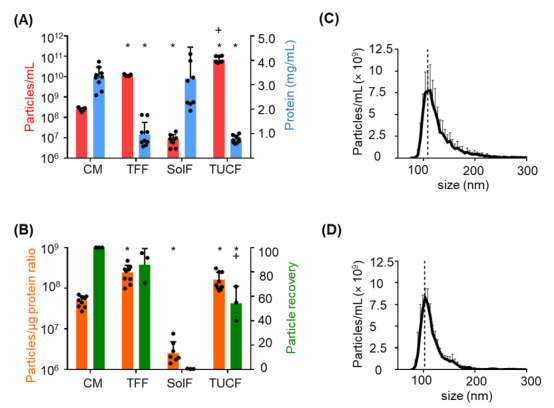
Monitoring iPSC-EV purification efficacy. (**A**) Particle concentration (red bars) as measured by tunable resistive pulse sensing (TRPS) and protein concentration (Bradford; blue bars) of conditioned media (CM), EVs after tangential flow filtration (TFF), cell-secreted soluble factors (SolF) separated in the TFF permeate, EVs ultra-centrifuged after TFF (TUCF). Mean ± SD of three independent experiments (* *p* < 0.05; compared with CM and (+ *p* < 0.05; compared with TFF). (**B**) The particle/µg protein ratio (orange bars) was calculated by dividing the total particle count by total protein amount. Mean ± SD of three independent experiments. Efficiency of EV production (particle recovery; green bars) comparing the number of particles in the starting material (CM = 100%) to the particle number after TFF, in the SolF and after TUCF. (**C**,**D**) Illustration of particle size after (**C**) TFF and (**D**) TUCF purification. Lines represent mean data from three independent experiments ± SD. Dotted line indicates particles size peak mode of 109 nm in (**C**) and 103 nm in (**D**).

**Figure A1:**
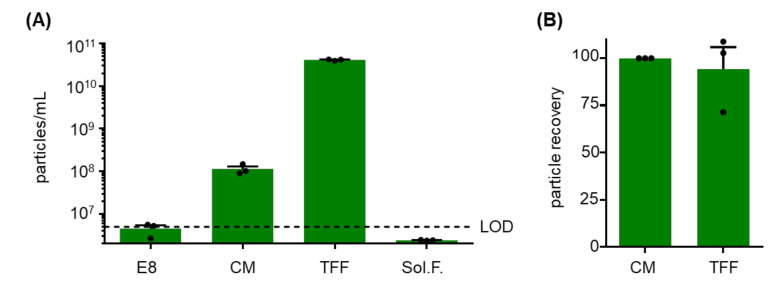
Preliminary results of 3D large scale culture for EV production. (**A**) Particle counts and (**B**) recovery in a pilot experiment using 3D human iPSC cultures in the DASbox Mini Bioreactor System (DASGIP-Eppendorf) as measured by tunable resistive pulse sensing (TRPS). Serum-free defined E8 medium showed particle counts at or below the limit of detection (LOD; dotted line) of the measurement system. This enabled accurate analysis of iPSC-derived particles, presumably representing extracellular vesicles, in iPSC-conditioned medium (CM) before and after enrichment by tangential flow filtration (TFF) with particle recovery comparable to the main study (technical triplicate analysis).

Pooled conditioned medium contained 2.516 × 10^8^ ± 0.422 × 10^8^ (mean ± SD) particles with a protein content of 2.971 ± 0.263 mg/mL (mean ± SD). TFF resulted in a significant particle enrichment and significant depletion of protein. The soluble factors in the TFF retentate contained mean < 10^7^ particles/mL and a protein concentration comparable to the conditioned medium. Further significant particle enrichment was realized by ultracentrifugation with further depleting protein (Figure 2A). EV preparation purity as determined by the particle/protein ratio was significantly increased by TFF and by TUCF. Particle recovery was significantly higher after TFF compared to TUCF (Figure 2B). Mean particle size was 109 nm and 103 nm after TFF and TUCF, respectively (Figure 2C,D). In a pilot experiment, we tested whether 3D culture of human iPSCs in an established bioreactor system resulted in equivalent EV secretion. Comparable iPSC-EV counts were detected in conditioned medium and after TFF enrichment with an appropriate recovery (Figure A1) compared to 2D conditions (Figure 2).

### 2.3. Marker Identity of iPSC-EVs

**Figure 3:**
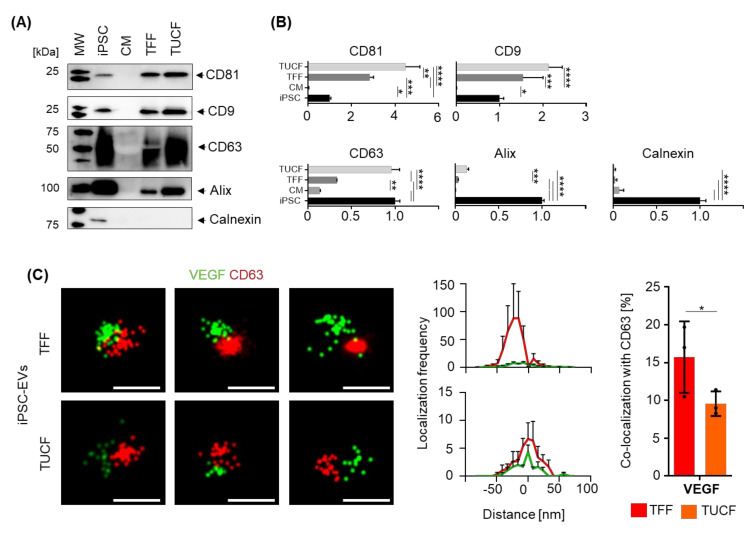
Characterization iPSC-EVs. (**A**) Human iPSC EVs in conditioned medium (CM), after enrichment by tangential flow filtration (TFF), and sub-sequent ultracentrifugation (TUCF) were compared to their parental iPSCs by immunoblotting for tetraspanins CD81, CD9 and CD63, for the EV marker alix and the cell-compartment contamination marker calnexin. Representative Western blots of two independent experiments. (**B**) Quantification of band intensity normalized to total protein loaded. Bars represent results from three independent replicates (**** *p* < 0.0001, *** *p* < 0.001, ** *p* < 0.01, * *p* < 0.05). (**C**) Representative super-resolution microscopy images of TFF- vs. TUCF-enriched iPSC-EVs showing co-localization of VEGF (green), and CD63 tetraspanins (red) as indicated. Scale bar 100 nm. Significantly more VEGF signal co-localization was determined on CD63 tetraspanin-labeled iPSC-EVs isolated by TFF than after TUCF as indicated.

We used immunoblotting to identify the bulk iPSC-EV identity according to MISEV2018 guidelines [19]. Tetraspanins CD81, CD9, CD63 and the endosomal targeting protein alix, as well as the endoplasmic reticulum integral protein calnexin, were detected in iPSCs but not in the conditioned medium as expected. TFF-enriched human iPSC-EVs were characterized by the presence of tetraspanins and alix. Significant further enrichment of tetraspanins CD81 and CD63 and alix was detected in iPSC-EV preparations after TUCF. EV purity after TFF and TUCF was confirmed by lack of calnexin reactivity compared to a positive signal in iPSCs (Figure 3A,B). We used super-resolution direct stochastic optical reconstruction microscopy (dSTORM) to further characterize iPSC-EVs at the single-vesicle level. The proportion of EVs that also displayed a co-localized VEGF signal was significantly higher among TFF-enriched iPSC-EVs (15.73% ± 4.73%; mean ± SD) compared to TUCF-enriched iPSC-EVs (9.57% ± 1.63%; mean ± SD). We observed mean 92 and mean 12 iPSC-EVs co-localizing VEGF with CD63 signals among mean 531 and mean 122 TFF-enriched and TUCF-enriched iPSC-EVs by dSTORM analysis (Figure 3C,D).

### 2.4. Efficacy of Hypoxia

**Figure 4:**
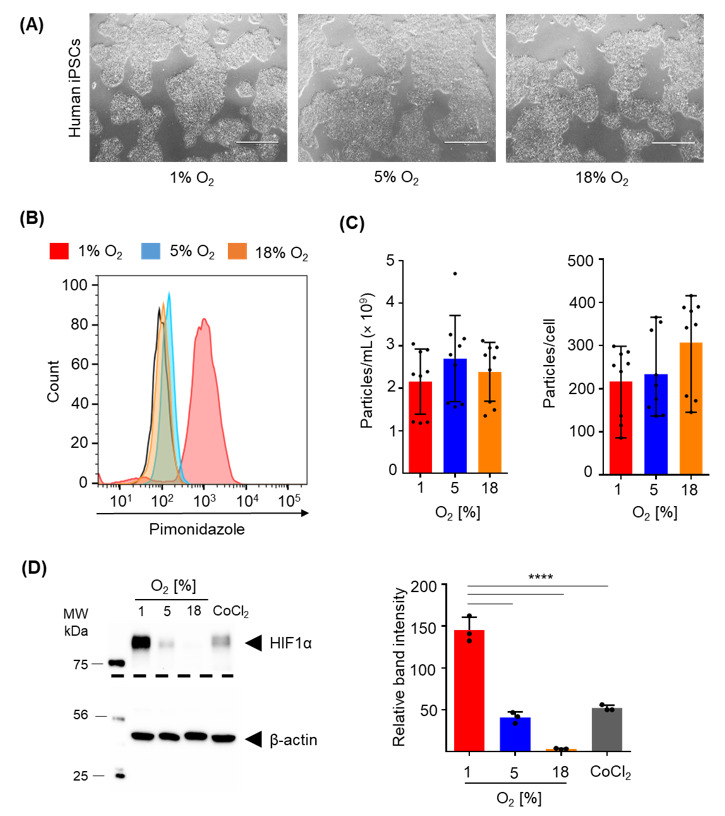
Conditioning of iPSCs at different oxygen levels during EV production. (**A**) Representative images of iPSC cultures at different oxygen levels as indicated. Scale bar 1000 µm. (**B**) Formation of oxygen adducts measured by flow cytometry with the hypoxyprobe pimonidazole. Color code as indicated; black line shows unstained sample. (**C**) Particle concentration and particles secreted per cell per 24 h, by human iPSCs at indicated oxygen levels. Bars represent replicates from three independent experiments. (**D**) Representative immunoblotting for HIF-1α levels in human iPSCs at different oxygen levels. CoCl_2_ was used as a positive control for HIF-1α stabilization. Protein quantification (relative band intensity) normalized to total protein loaded (right graph in (**D**)). Bars represent mean ± SD results from three independent replicates (**** *p* < 0.0001).

We tested different oxygen levels during iPSC culture because the level of oxygen required for effective hypoxic conditions for different cell types is a matter of debate [26]. Starting from equal numbers of oligoclonal human iPSCs colonies cultured under 1%, 5% and 18% air oxygen condition in six-well plates, we generated 1.253 × 10^6^ ± 0.514 × 10^6^, 1.618 × 10^6^ ± 0.670 × 10^6^ and 1.069 × 10^6^ ± 0.495 × 10^6^ iPSCs, respectively, within four days. Cultures showed comparable morphology with typical colonies that displayed smoothly defined edges in advance of confluence irrespective of oxygen conditions (Figure 4A).

The hypoxyprobe pimonidazole, known to covalently bind to thiol-containing proteins in hypoxic cells [27], produced a profound homogenous signal measured by flow cytometry at the single-cell level only after culture at 1% oxygen. A minor hypoxia response was observed after 5% oxygen culture and no response at ambient air, serving as control (Figure 4B). No significant difference in particle concentration, representing EV release, was observed in the conditioned medium irrespective of the oxygen level. Additionally, after correction for differences in cell proliferation, no significant difference in the number of particles secreted per cell was found (Figure 4C). Immunoblotting confirmed flow cytometry results by showing significant stabilization of the hypoxia-inducible transcription factor HIF-1α at 1% compared to 5% or 18% oxygen culture of human iPSCs (Figure 4D).

### 2.5. Angiogenic Potential of Human iPSC-EVs

**Figure 5:**
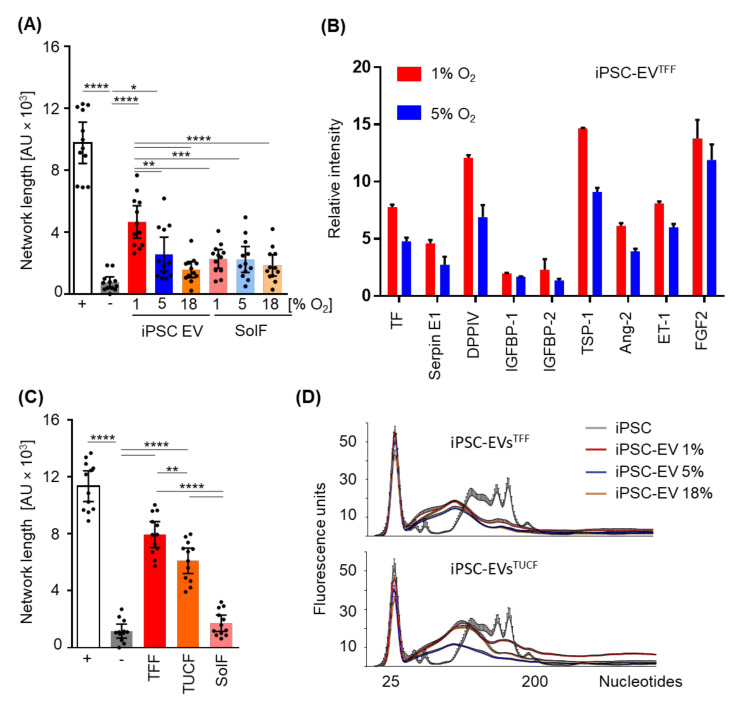
Angiogenic potential of iPSC-EVs obtained after iPSC conditioning at different oxygen levels. (**A**) Total length of the endothelial networks in the absence (white bar, positive control; grey bar negative control) or presence of iPSC EVs and corresponding soluble factors (SolF) obtained at defined oxygen levels as indicated and separated by tangential flow filtration (TFF), respectively (**** *p* < 0.0001, *** *p* < 0.001, ** *p* < 0.01, * *p* < 0.05). (**B**) Angio-profiler Western blot array of TFF-enriched iPSCs (duplicate analysis on one randomly selected EV preparation) normalized by equal EV number input. (**C**) Total length of endothelial networks in the absence or presence of iPSC-EVs derived from hypoxic iPSC culture and produced by TFF or TFF plus ultracentrifugation (TUCF) or by adding EV-depleted soluble factors (SolF) obtained as TFF retentate from 1% oxygen culture conditions. Bars represent pooled results from three independent experiments (**** *p* < 0.0001, ** *p* < 0.01). (**D**) Electropherogram analysis of different RNA samples isolated from TFF- (top) and TUCF-purified EVs (bottom) derived from different oxygen conditions as indicated and run on a Bioanalyzer RNA pico chip (mean ± SD; n = 3). Grey curves represent the small RNA profile of the same parental iPSCs in both histograms for comparison.

**Figure A2:**
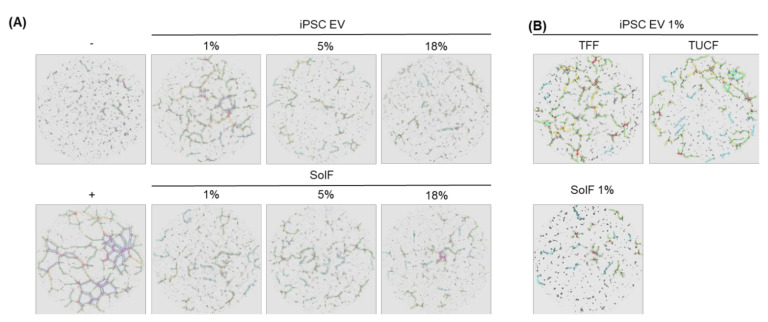
Angiogenic potential of iPSC-EVs. (**A**) Representative endothelial networks in the presence of iPSC EV obtained from iPSC cultures at different oxygen level as indicated compared to negative (−) and positive (+) control, respectively. (**B**) Endothelial networks in the presence of iPSC-EV derived from hypoxic 1% oxygen culture and isolated by TFF or TUCF and compared to addition of equivalent volume of soluble factors (SolF) corresponding to the TFF preparation. Representative images from three independent experiments showing network detection by imageJ plugin Angiogenesis Analyser.

TFF-enriched iPSC-EVs derived from large-scale human iPSC cultures conditioned at different oxygen levels were tested for their capacity to replace optimized pro-angiogenic factors, i.e., stimulate vascular network formation, in a well-characterized angiogenesis assay in vitro. In the absence of otherwise established pro-angiogenic factors, iPSC-EVs derived from 1% oxygen cultures induced angiogenesis significantly more potent than those derived from 5% or 18% oxygen-conditioned iPSC culture. Interestingly, the hypoxic iPSC-derived EVs were significantly more potent than corresponding soluble factor fractions, indicating the presence of pro-angiogenic proteins or other active components associated with iPSC-EVs (Figure 5A and Figure A2). We therefore compared iPSC-EVs derived from 1% and 5% oxygen cultures for their protein profile using a Western blot-based angio-profiler array in two selected iPSC-EV preparations. A constantly higher level of angiogenesis-inducing proteins was found in TFF-purified EV preparations despite equivalent protein input.

To address the question if the separation method influenced iPSC function, in addition to the oxygen level during culture, we next compared TFF-enriched iPSC-EVs with their corresponding soluble factors (derived from the TFF retentate) and TUCF-purified iPSC-EVs for their angiogenic potential. TFF-enriched hypoxic culture-derived iPSC-EVs (1% oxygen) were significantly more potent than their corresponding soluble factors and also significantly more potent than further TUCF-purified iPSC-EVs (Figure 5C). MicroRNAs and additional small RNA species were detected in both TFF- and TUCF-enriched iPSC-EVs. Further detailed analysis will be required to identify the precise small RNA profile in iPSC-EVs obtained in hypoxic conditions. The results obtained so far therefore build the basis for future studies aiming to understand the differential contribution of EV protein as compared to RNA cargo to iPSC-EV function.

## 3. Discussion

This study illustrates that both the cell culture conditions and EV isolation modalities can significantly affect the function of human iPSC-EVs. We first demonstrated that conventional 2D large-scale feeder-free human iPSC propagation on an extended growth area of 2528 cm^2^ per four-layered cell factory, as established previously for bone marrow stromal cells [28], is sufficient to harvest at least 2.7 L of EV-containing conditioned medium per cell factory within five days. The canonical human iPSC phenotype was maintained during large-scale culture on matrigel as demonstrated by flow cytometry. The least laborious way to obtain >2 L iPSC-EV-containing conditioned medium would be daily harvest of the otherwise discarded ‘consumed’ cell culture medium (e.g., 12 mL per six-well plate per day for 14 weeks, cryopreserved at −80 °C until pooling and further processing). The more sophisticated strategy would be a scalable 3D bioreactor-based cell culture [29,30]. In a pilot experiment, we obtained 2.0 L of iPSC-derived conditioned medium collected from two parallel cultures within four days from bioreactor culture-derived conditioned medium. We conclude that the more scalable 3D culture of human iPSCs may be the preferred way for future iPSC-EV manufacturing. The 2D culture using standard equipment still offers definitive advantages at laboratory scale for research purposes. A follow-up in-depth analysis comparing EV production quantity, quality and functionality of selected iPSC clones under defined 2D vs. 3D oxygen-controlled conditions is currently in preparation.

There are two general strategies to obtain EV-conditioned media for research and therapy based on using particle-reduced vs. particle-rich media depending on the cell culture model. Particle-rich media, due to supplementation with human platelet lysate or fetal bovine serum, were instantly used to acquire bone marrow stromal cell-derived EVs for cell-free treatment of graft-versus-host disease [31]. Alternatively, pre-existing particles could be depleted before initiation of cell culture [32]. Chemically defined serum-free medium offers the advantage of a lower initial particle count, thus enabling a more precise quantification of EVs secreted by donor cells into the conditioned medium over time. We observed a significant mean >ten-fold increase in particle counts in iPSC-conditioned mTeSR medium in this study, arguing that >90% of the final particle load in the conditioned medium would be cell-derived EVs. Significant enrichment of these iPSC-EVs by TFF and TUCF also allowed investigation of the EV-depleted soluble factors of the iPSC secretome. The EV enrichment and soluble protein separation process, respectively, was documented as significant increases in the particle/protein ratio. TUCF was more efficient than TFF for particle enrichment at the expense of particle recovery. The particle size was not affected by either method. Based on the application envisioned, it needs to be determined, whether the still protein-rich vesicular secretome fraction obtained by TFF is more efficient than the more purified EV product after TSEC [33]. For research purposes, higher purity of EVs may help to stratify the mode of action of cell secretomes into EV-based vs. soluble factor-mediated pathways. In our study, we did not address the question whether small exomeres may reside within the soluble factor fraction. We demonstrated significant stepwise iPSC-EV enrichment following MISEV2018 guidelines [19]. This builds the basis for future more mechanistic experiments. Using dSTORM super-resolution microscopy, we could demonstrate that the EV isolation method has an impact on EV quality as evidenced by a reduced proportion of VEGF-bearing EVs after TUCF as compared to the less stringent TFF isolation. This translated to a higher angiogenic potential of TFF-enriched iPSC-EVs in our study.

We further compared the impact of different prototypic oxygen levels during iPSC culture on iPSC-EV quantity, quality and function. We selected 1% air oxygen and 12–16 h medium equilibration in advance of cell culture in a hypoxic workstation closed system to mimic hypoxia during iPSC culture. Air oxygen at 5% representing the stem cell niche conditions was compared to standard cell culture, both in a nitrogen-controlled incubator system at 5% CO_2_ in humidified air. Interestingly, no significant difference was found for EV production as concluded from comparable particle counts after five days iPSC culture at the three oxygen conditions.

The optimum oxygen level during cell culture is a matter of debate [26]. It is meanwhile widely accepted that 21% ambient air oxygen, corresponding to approximately 18% O_2_ in a 5% CO_2_-containing incubator air mix, does not represent the universal normoxia for every cell type [34]. Oxygen was recognized as a particularly critical component of the stem cell niche with levels ranging from 1–8% being described as optimum for maintaining stemness of stromal cells and pluripotency of iPSCs [35]. We practice human PSC standard culture at 5% air oxygen based on accumulated experience and published evidence [36]. We further standardized 2D cultures by using precisely 0.2 mL of supplemented culture medium per cm^2^ (e.g., 505 mL per cell factory) for creating a standardized air oxygen diffusion length. Physical parameters like diffusion length and dissolved oxygen are as important as physio-pathological considerations [26]. We measured 40% dissolved O_2_ at 8.4% air oxygen in a pilot experiment set up to determine iPSC-EV production in a more scalable 3D bioreactor. We were not able to monitor dissolved oxygen levels in our 2D cultures. The cell density known to impact the hypoxia response was kept sub-confluent under 2D and defined by input cell concentration used under 3D conditions.

Pimonidazole staining and significant HIF-1α stabilization demonstrated effective hypoxia response in iPSCs cultured at 1% O_2_ under our standardized conditions. A significantly lower level of HIF-1α stabilization and minute levels of pimonidazole binding were observed at 5% O_2_. This is in accordance with previous findings demonstrating that HIF-2α and Notch signaling were key mediators of the stem cell response to mildly reduced oxygen levels in the niche [35]. Interestingly, iPSC-EVs derived from 1% O_2_ cultures were significantly more potent than other iPSC-EVs in inducing vascular network formation in vitro. The observation that 5% O_2_ culture-derived iPSC-EVs also induced significantly more angiogenesis is in accordance with previously published in vivo data on murine heart revascularization [23]. The iPSC-secreted soluble factors under all three O_2_ conditions tested were found to be significantly less efficient inducers of angiogenesis than the 1% oxygen conditioned iPSC-EVs.

We selected undifferentiated human iPSCs as EV donor cells because they can be propagated under defined conditions to virtually unlimited quantity. Recent data also indicate that iPSCs of mice and man can support heart reperfusion after injury and protect endothelial cells from diabetic angiopathy [23,24]. Several other recently published studies used EVs derived from more mature iPSC progeny (Table A1). EVs of iPSC-derived endothelial cells were found to promote angiogenesis after mouse hindlimb ischemia [37]. Stromal cells generated from iPSCs can produce EVs that induce angiogenesis after skin injury [38], limb ischemia [39] and osteonecrosis [40]. Additionally, iPSC-derived cardiomyocytes [41] and cardiac progenitors [42] showed pro-angiogenic potential contributing to cardiac recovery post-infarction. Most of these studies used ultracentrifugation for EV purification (Table A1), indicating that EV cargo rather than contaminating secreted proteins, which are depleted by ultracentrifugation, mediate the angiogenic effect. Our study clearly demonstrated scalability of TFF for producing large amounts of pro-angiogenic human iPSC-EVs according to MISEV guidelines (Table A2). Further purification by TSEC reduced the pro-angiogenic effect of our iPSC-EVs. The pro-angiogenic protein cargo was constantly higher in iPSC-EV preparations obtained from hypoxic 1% O_2_ iPSC cultures through TFF. Based on these data we can just speculate that hypoxia-induced proteins contribute to the observed effect. Another limitation of the current study relates to the fact that the analysis of the molecular mechanisms underlying the pro-angiogenic effects of iPSC-EVs obtained during hypoxic iPSC culture is still pending. In addition to hypoxia-induced proteins, microRNAs within EVs represent prime candidates for mediating therapeutic re-vascularization [43]. In a first attempt, we confirmed enrichment of small RNA species in our EV preparations. A more detailed iPSC-EV RNA profiling is planned once the large-scale manufacture conditions have been defined.

## 4. Materials and Methods

### 4.1. Human iPSC Culture under Different Oxygen Levels

Human iPSCs were generated by reprogramming bone marrow stromal cells or umbilical cord blood cells obtained with permission from the Institutional Review Board of the Medical University of Graz ((protocols EK 19–252, EK 21–060) and the Ethics Committee of the province of Salzburg (protocol 415-E/1776/4-2014) [44,45]. Human stromal cell samples were collected from healthy volunteers after written informed consent according to the Declaration of Helsinki. For genetic reprogramming, using a non-integrative Sendai viral vector kit (CytoTune™-iPS Sendai Reprogramming Kit encoding for Oct4, Sox2, KLF4 and c-Myc, Life Technologies, Carlsbad, CA, USA) was used and performed at the Harvard Stem Cell Institute (HSCI) iPSC Core Facility (Cambridge, MA, USA) adapted from an established protocol as previously described [7,46]. Human iPSCs were cultured in six-well plates (Corning, Corning, NY, USA) coated with Matrigel^®^ (Corning, USA) in mTeSR™1 medium (STEMCELL Technologies, Vancouver, CB, Canada). If not indicated differently, air oxygen conditions during cell culture were 5%. For EV production, 80% confluent iPSC colonies were expanded from six-well format to one T225 flask and, after reaching 80% confluency again, to one four-layered cell factory (2528 cm^2^, CF4, Thermo Fisher Scientific, Waltham, MA USA) in chemically defined particle-poor medium (mTeSR™1, STEMCELL Technologies). The total volume of conditioned medium (CM; 450 mL/CF4) was collected daily and replaced by fresh medium, starting when colonies reached 50% confluency (day 3–4) until 80% confluency (day 8). For evaluating the influence of different oxygen conditions on EV quantity and quality, CM was harvested from iPSC cultures at 1%, 5% or 18% oxygen in different culture vessels. Culture at 1% oxygen was performed in a closed system hypoxia workstation (H35 Hypoxystation; Don Whitley Scientific, Victoria Works, UK), while 5% or 18% oxygen was maintained in a nitrogen-controlled cell-culture incubator (Binder, Tuttlingen, Germany).

### 4.2. 3D Human iPSC Culture

A pilot experiment evaluating EV release under 3D culture conditions was performed. For this experiment, aggregate-based suspension culture of human iPSCs was conducted as published [30]. In short, a single cell suspension of human iPSCs was inoculated in the DASbox Mini Bioreactor System (Eppendorf, Hamburg, Gemany) in E8 medium supplemented with Y-27632 at a cell density of 0.5 × 10^6^ cells/mL. Cells were cultivated at 37 °C, stirred at 80 revolutions per minute (rpm), headspace aerated with 0.9 sL/hour, dissolved oxygen controlled at 40% (equivalent to 8.4% air oxygen) and pH controlled at 7.1. No media changes were performed for 24 h after single-cell inoculation. Subsequently, the culture medium was replaced constantly in perfusion mode at increasing flow rates, while the cells were retained in the bioreactor.

### 4.3. EV Purification

Immediately after collection, the CM was centrifuged (300× *g* for 5 min and 3000× *g* for 10 min) to remove cells and cell debris, respectively, and the supernatant was stored at −80 °C. For EV enrichment and purification, total CM (2700 mL) of one consecutive culture was pooled after thawing. The CM was concentrated 150-fold using a 300 kDa cut-off hollow fiber modified polyethersulfone (mPES) membrane filter column operated on a KR2i TFF System (Repligen, Waltham, MA, USA). The permeate fraction (<300 kDa) was collected (now termed ‘soluble factors’). To deplete proteins, the EVs concentrated in the system were subsequently iso-volumetrically washed with twice the start volume in 0.9% NaCl buffered with 10 mM HEPES and were termed TFF-EVs. For further protein depletion, TFF-EVs were diluted 1:10 in 0.9% NaCl buffered with 10 mM HEPES and subjected to two consecutive cycles of ultracentrifugation (UCF, 100,000× *g* for 90 min) in a WX80 ultracentrifuge equipped with a TH-641 swing rotor (Thermo Scientific). The pellet was resuspended in 0.9% NaCl buffered with 10 mM HEPES and was termed TUCF-EVs.

### 4.4. Determination of EV Concentration and Size via TRPS

Concentration and size of particles were determined by TRPS using a qNano Gold analyzer (Izon, Christchurch, New Zeeland) equipped with an NP150 nano-pore for a size range between 70 and 450 nm. Dulbecco’s phosphate buffered saline (DPBS; Sigma-Aldrich, St. Louis, MO, USA) with 0.05% Tween 20 filtered through a 0.22 µm syringe filter (Ahlstrom-Munksjö, Helsinki, Finland) was used as measurement electrolyte. Samples were measured in 1:1, 1:20 or 1:50 dilutions in triplicate using a fixed pressure of 10 mbar and current of 120 nA.

### 4.5. Analysis of EV Markers and Proteins by Western Blot Analysis

The protein concentration of EV and CM samples was determined by Bradford assay (Bio-Rad, Hercules, CA, USA) according to manufacturer’s recommendations. OD was measured at 595 nm analyzed in a plate reader SPARK 7 (Tecan, Grödig, Austria). For SDS page separation, EV and CM samples were mixed with 4× Laemmli buffer (Bio-Rad) for non-reducing conditions, used for tetraspanins (CD9, CD63 and CD81), or with Laemmli buffer containing 50 µM dithiothreitol (DTT), as reducing agent for all other markers. Samples were run on a 4–20% SDS-PAGE Gel (Bio-Rad) in a protean mini system (Bio-Rad). To estimate the molecular weight of proteins for proper identification of Western blot bands, a molecular weight marker (Precision Plus protein dual Xtra prestained protein standard, Bio-Rad) was used. Proteins were transferred to polyvinylidine difluoride (PVDF) membranes using the Trans-Blot Turbo Transfer system (Bio-Rad). After blocking membranes with tris-buffered saline (TBS) containing 0.05% Tween 20 and 2% bovine serum albumin (BSA), specific proteins were detected using monoclonal antibodies against CD63 (0.05 µg/mL, clone TS63, Thermo Fisher, USA), CD81 (0.04 µg/mL, clone 1.3.3.22, Thermo Fisher), CD9 (0.5 µg/mL, clone IVA50; Invitrogen, Carlsbad, CA, USA), alix (0.8 µg/mL, clone 3A9; Cell Signaling, Danvers, MA, USA) and calnexin (0.026 µg/mL; Cell Signaling). Detection was performed using horseradish peroxidase (HRP)-conjugated secondary antibodies (rabbit anti-mouse IgG, A27025, Thermo Fisher; mouse anti-goat 205-035-108, Jackson Laboratories, Bar Harbor, ME, USA; or polymer goat-anti-rabbit, K4002, DAKO EnVision, Agilent, Santa Clara, CA, USA). Specific bands were visualized and quantified after incubation with Clarity Western ECL substrate (Bio-Rad) using a ChemiDoc^TM^ Imaging system and Image Lab software (both Bio-Rad). 

### 4.6. Flow Cytometry

Human iPSCs (n = 3) were prepared for staining by washing in PBS (0.5% BSA) and blocking in 5% sheep serum (Sigma-Aldrich). After preconditioning with 1%, 5% and 18% oxygen conditions iPSCs were analyzed for pluripotency markers using surface and intracellular antibodies SSEA4-BUV395 (2 µg/mL, clone MC813-70), Tra-1-81-AF647 (0.12 µg/mL clone TRA-1-81), Nanog-PE (2.5 µg/mL, clone N31-355; all from BD, Franklin Lakes, NJ, USA) and Oct4 PE (2.5 µg/mL, clone 3A2A20; Biolegend, San Diego, CA, USA). For the hypoxyprobe assay, 1 × 10^6^ cells were incubated 2 h prior to harvest with 100 µM pimonidazole (Sigma, USA) or with dimethyl sulfoxide (DMSO) as control in 6-well plates under given oxygen conditions. After harvest, iPSCs were stained with anti-pimonidazole-FITC antibody (6 µg/mL, clone 4.3.11.3, HPI, Burlington, MA, USA). Staining with fluorochrome-labeled antibodies was performed for 20 min at 4 °C. Appropriate isotype matched antibodies were used in the same concentration as the tested antibodies as negative controls (IgM-AF647 clone MM-30 for Tra-1-81, Biolegend; IgG3-BUV395 clone J606 for SSEA-4, BD; IgG1-PE clone MOPC-21 for Nanog, BD; IgG2b-PE clone MPC-11 for Oct4, Biolegend; IgG1 clone ×40 for pimonidazole, BD). Viability of cells was determined by staining with fixable viability dye FVD520 (1:100, eBioscience, San Diego, CA, USA) and a minimum of 10,000 FVD520-negative viable cells was acquired. Flow cytometry was performed with a five-laser BD LSR-Fortessa (BD), BD FACSDiva Software 8.0.1 Firmware version 1.4. Analysis of data was performed with FlowJo 10.7.1 (BD).

### 4.7. Immunomodulation Assay (IMA)

**Figure A3:**
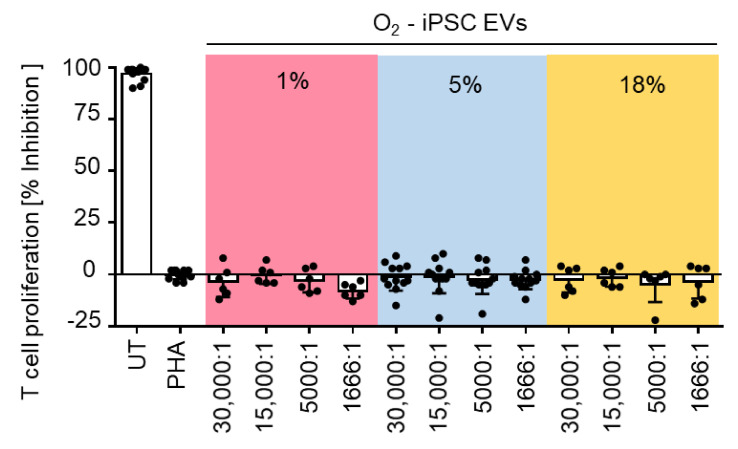
No inhibition of T cell proliferation by iPSC EV generated from human iPSCs at. Mitogenesis of T cells was induced by adding phytohemagglutinin (PHA) and compared to the proliferation of untreated (UT) T cells. Human iPSC-EVs that were generated in iPSC cultures at different oxygen levels and analyzed in triplicates per experiment (iPSC-EVs 1%, n = 2; iPSC-EVs 5%, n = 4; iPSC-EVs 18%, n = 2) did not inhibit the PHA-induced T cell mitogenesis.

The hypothetic effect of iPSC EVs on the immune response was analyzed using a T-cell proliferation assay as published earlier [47,48]. In brief, peripheral blood mononuclear cells (PBMCs) were isolated and pooled from 10 individual donors before labelling with carboxyfluorescein succinimidylester (2 µM CSFE; Sigma-Aldrich) and cryopreservation in appropriate aliquots for later use. These pre-labeled PBMCs (300,000 per flat-bottomed 96-well plate) were stimulated with 5 µg/mL phytohemagglutinin (PHA, Sigma-Aldrich). Assays were incubated with EVs dose dependently at three-fold serial dilution in a ratio of 15,000:1, 5000:1, 1666:1, or 555:1 for four days (n = 2–4). For staining, cells were prepared by washing in PBS. Cells were incubated with a CD3-APC antibody (2.5 µg/mL, clone SK7, BD). Acquisition of minimum 10,000 positive events was performed on the flow cytometer Gallios (Beckman Coulter, Brea, CA, USA) and analyzed by Kaluza software 1.3 (Beckman Coulter). The percentage of proliferating T cells was determined as the fraction of viable CD3 positive cells with reduced CFSE staining compared to non PHA-stimulated cells. In contrast to EVs from various types of stromal cells [49], the iPSC-EVs did not inhibit T cell mitogenesis (Figure A3).

### 4.8. Super-Resolution Microscopy

For single EV surface marker analysis, EV samples (50 µL; n = 3) were coated onto 18-well µ-slides (glass bottom, Ibidi, Gräfelfing, Germany). After incubation at 4 °C overnight, the slides were labelled with CD63-AF647 (10 µg/mL, clone H5C6, BD) and VEGF-AF488 (5 µg/mL clone 23410, RD Systems, Abingdon, UK) antibodies, and analyzed by super resolution microscopy (Nanoimager S, Oxford Nanoimaging, Oxford, UK). Images were taken in dSTORM mode using 30% laser power for the 640 nm and 50% laser power for the 488 nm laser, and 2500 images per channel for localization mapping. Data were processed with the collaborative discovery (CODI) online analysis platform using the drift correction pipeline version 0.2.3. Clustering analysis was performed for 16–400 localizations within a 10–1000 nm radius. Co-localization was defined by a minimum number of localizations for CD63/VEGF signals within a distance of 180 nm.

### 4.9. In Vitro Angiogenesis Assay

EV preparations and soluble factors from iPSC cultures were used to assess their angiogenic potential using an ECFC network formation assay on matrigel as previously described [11]. Umbilical cord blood (UCB)-derived ECFCs were cultured in EGM-2 endothelial cell growth basal medium (EBM-2, Lonza, Basel, Switzerland) supplemented with 5 mM (N2)-L-alanyl-L-glutamin (Dipeptiven^®^, Fresenius Kabi, Linz, Austria), 2 U/mL preservative-free heparin (Biochrom, Berlin, Germany), 100 U/mL penicillin and 0.1 mg/mL streptomycin (Sigma-Aldrich) and with EGM-2 SingleQuot supplements (hydrocortisone, hFGF, VEGF, IGF, EGF and ascorbic acid; Lonza). Fetal bovine serum was replaced by 10% v/v of pooled human platelet lysate (HPL) as previously described [4,50]. ECFCs were starved prior to the assay for 18 h in growth factor-free endothelial cell basal medium (EBM-2) supplemented with 4% human serum albumin (Fresenius Kabi). For the network formation assay starved ECFCs were seeded (6000 cells/well) on a reduced growth factor basement membrane matrix (Geltrex, Thermo Fisher) in an angiogenesis 96-well µ-plate (Ibidi) in non-growth factor supplemented endothelial cell growth medium (EBM-2, Lonza). To estimate the influence of EVs or soluble factors on ECFC network formation, EV preparations were added in an EV to ECFC ratio of 10,000:1 and compared to the volume equivalent of soluble factors added to ECFCs. SingleQuot-supplemented EGM-2 was used as positive control, whereas the basal medium EBM-2 supplemented with 4% v/v of human serum albumin (Fresenius Kabi) served as negative control. Images were taken every hour for 16 h on an Eclipse Ti inverted microscope (Nikon, Tokyo, Japan) equipped with a custom-build live cell incubation system (Oko Lab, Pozzuoli, Italy) using a 4× objective. Images were processed with the NIS Elements Advanced Research package analysis software (Nikon). Total geltrex areas were cut out of raw images, homogenized (strength 16), and subjected to intensity equalization over different positions. Afterwards, pictures were sharpened slightly and denoised (advanced denoising 11.0). Exported images were cut at the diameter of 1200 pixels to remove edges of the plate and the contrast was enhanced. Processed pictures were analyzed by Image J software version 1.52p [51] and angiogenesis analyzer plugin to automatically determine the network and total length of tube-like structures [52].

### 4.10. Analysis of EV’s RNA Cargo

To estimate and characterize the RNA cargo of iPSCs and differentially prepared EVs, total RNA was extracted using the RNA/DNA purification kit (Norgen Biotek, Thorold, ON, Canada) and stored at −80 °C. For analysis, 1 μL RNA was loaded on a RNA 6000 Pico Chip (Agilent), scanned in the 2100 Bioanalyzer (Agilent) according to the manufacturer’s instructions and analyzed by Agilent 2100 Expert software (version B.2.10 SI764). 

### 4.11. Angiogenesis Profiler Arrays

Angiogenesis-related proteins of iPSC-EVs isolated by TFF or TUCF were measured using proteome profiler arrays (Proteome Profiler Human Angiogenesis Array Kit, ARY007, R&D Systems) according to the manufacturer’s protocol with loading 100 µg protein per membrane. Specific membrane dots were visualized and quantified using a ChemiDoc™ Imaging system and Image Lab software (both Bio-Rad).

### 4.12. Statistics

Data were compared by analysis of variance (ANOVA) using Tukey’s post-test correction in GraphPad Prism version 7.03 (GraphPad Software, San Diego, CA, USA). Differences were considered statistically significant with a *p*-value < 0.05.

## 5. Conclusions

This study shows that both the oxygen conditions during cell culture and the EV preparation strategy (e.g., TFF with or without subsequent ultracentrifugation) affect iPSC-EV function. We conclude that well-defined conditions need to be established for the manufacturing of particularly potent iPSC-EV-based therapeutics.

## Appendix A

**Table A1:** Studies evaluating the angiogenic potential of iPSC-derived EVs.

EV Source	Intervention	EV Isolation	Outcome	Reference
hiPSC	In vitro high glucose challenge	UCF	iPSC-exosomes protected endothelial cells from high glucose-induced senescence	[24]
hiPSC	Growth factor depriva-tion during angiogenesis	TFF	EVs from both cell types enhanced growth factor-induced in vitro angiogenesis	[53]
Murine iPSC	Mouse myocardial infarction	UCF	Promoted in vitro angiogenesis and improved cardiac recovery post-infarction in vivo	[23]
Rhesus macaque iPSC	Macaque skin wound healing	Exo-quick + UCF	In vivo wound healing with increased number of capillaries	[54]
hiPSC-MSCs (stromal cells)	Cranial lesions in osteopenic mice	UF + DGC + UF	Promoted in vivo vessel formation in calvaria lesions	[49]
hiPSC-MSCs (stromal cells)	Rat stroke model	UCF	Promoted in vitro and in vivo angiogenesis	[55]
hiPSC-SMPCs	Cardiotoxin injury	UF + SEC + UF	EVs promoted in vitro angiogenesis	[56]
hiPSC-SMCs	Mouse hindlimb ischemia	UCF	iPSC-SMC exosomes promoted endothelial migration and proliferation	[57]
hiPSC-CMs	Angio- & scratch assay	Exo-quick	EVs promoted proliferation & angiogenesis	[41]
Rat iVPC (vascular progenitor cells)	Mouse hindlimb ischemia	UF + SEC	EVs romoted angiogenesis in vitro and in vivo	[58]
hiPSC-CM; -EC, -SMCs	Swine myocardial infarction	Exo-quick	EVs promoted reperfusion after infarction	[59]
hiPSC-CMPs	Mouse myocardial infarction	UCF	EVs induced CM proliferation & outperformed cells for cardiac recovery post-infarction	[42]
hiPSC-ECs	Mouse limb ischemia	UCF	Promoted angiogenesis/blood perfusion	[37]
hiPSC-MSCs (stromal cells)	Mouse skin injury	UCF + UF	Promoted wound healing, fibroblast-collagen synthesis and angiogenesis	[60]
hiPSC-MSCs (stromal cells)	Steroid-induced rat osteonecrosis	UCF+ UF	Prevented bone loss and promoted angiogenesis	[40]
hiPSC-CMs	hIPSC-CM from HyperGEN cohort	UF + PEG + SEC	Delayed angiogenesis with hIPSC-CM-EVs from left ventricular hypertrophy patients	[61]
hiPSC-MSCs (stromal cells)	Mouse limb ischemia	UF + DGC + UF	Promoted angiogenesis/blood perfusion	[39]

**Abbreviations:** human iPSC (hiPSC), smooth muscle cells (SMCs), skeletal muscle progenitor cells (SMPCs), cardiomyocyte (CM), cardiomyocyte progenitors (CMPs), endothelial cells (ECs), ultracentrifugation (UCF), tangential flow filtration (TFF), ultrafiltration (UF), size-exclusion chromatography (SEC), polyethylene glycol precipitation (PEG).

**Table A2:** Adherence to MISEV2018 criteria for research with EV material.

Nomenclature				
Use of Generic Term EV for iPSC-EVs				
**Collection and Storage**				
Releasing cell information		cell type and origin	Human iPSC reprogramed from bone marrow	
		passaging	p28/p35	
		seeding density	cells/cm^2^	
		culture volume	500 mL	
		culture vessel	2528 cm^2^ (CF4)	
		oxygen level	1, 5 and 18%	
Culture conditions		culturing medium	mTeSR basal medium plus supplement (serum-free)	
		time of cultivation	4–8 days	
		harvesting medium	mTeSR basal medium plus supplement (serum-free)	
		time of cultivation	24 h	
		cell count at harvest	Mean 9.767 × 10^8^ cells	
Storage and recovery		conditioned medium	Storage temperature: −80 °C	
		EV preparations	Storage temperature: −80 °C/snap freeze	
**Isolation**				
Differential centrifugation		300× g for 5 min, SX4750, Allegra X-15R, Beckman Coulter 3000× g for 15 min, SX4750, Allegra X-15R, Beckman Coulter		
Ultrafiltration (TFF)		Tangential flow filtration pore size: 300 kDa; concentration factor 150, isovolumetric washing with twice start volume		
Ultracentrifugation (TUCF *)		100,000× g for 90 min, TH-641 swing rotor Thermo Scientific		
**Characterization**				
		**Parameter**	**Unit**	**Method**
Quantification	Size & concentration	particle number	1.15 × 10^10^ particles/mL (TFF) 6.95 × 10^10^ particles/mL (TUCF)	TRPS
		particle size	109 nm (TFF) 103 nm (TUCF)	TRPS
		particle/protein ratio	2.46 × 10^8^ particles/µg (TFF) 1.62 × 10^8^ particles/µg (TFF)	TRPS/Bradford
	Composition	protein content	578 mg/mL (TFF) 460 mg/mL (TUCF)	Bradford
		RNA content	3 pg/µL (TFF) 1 pg/µL (TUCF)	Bioanalyzer RNA 6000 pico
Identity		trans membrane proteins	CD9	WB
		trans membrane proteins	CD63	WB
		trans membrane proteins	CD81	WB
		cytosolic recovered in EVs	Alix-1	WB
		secretory pathway	Calnexin	WB
Visualization		TFF: super resolution microscopy (CD63/VEGF)		
		TUCF: super resolution microscopy (CD63/VEGF)		
**Functional Studies**				
Angiogenesis			effect of EVs (10,000:1) on endothelial network formation	
